# The Green Approach to the Synthesis of Bio-Based Thermoplastic Polyurethane Elastomers with Partially Bio-Based Hard Blocks

**DOI:** 10.3390/ma14092334

**Published:** 2021-04-30

**Authors:** Ewa Głowińska, Paulina Kasprzyk, Janusz Datta

**Affiliations:** Department of Polymer Technology, Faculty of Chemistry, Gdansk University of Technology, G. Narutowicza Street 11/12, 80-233 Gdansk, Poland; paulina.kasprzyk@pg.edu.pl (P.K.); janusz.datta@pg.edu.pl (J.D.)

**Keywords:** thermoplastic polyurethane elastomers, bio-based diisocyanates, diisocyanate mixtures, thermal properties, bio-based hard segments, thermomechanical properties

## Abstract

Bio-based polymeric materials and green routes for their preparation are current issues of many research works. In this work, we used the diisocyanate mixture based on partially bio-based diisocyanate origin and typical petrochemical diisocyanate for the preparation of novel bio-based thermoplastic polyurethane elastomers (bio-TPUs). We studied the influence of the diisocyanate mixture composition on the chemical structure, thermal, thermomechanical, and mechanical properties of obtained bio-TPUs. Diisocyanate mixture and bio-based 1,4-butanediol (as a low molecular chain extender) created bio-based hard blocks (HS). The diisocyanate mixture contained up to 75 wt % of partially bio-based diisocyanate. It is worth mentioning that the structure and amount of HS impact the phase separation, processing, thermal or mechanical properties of polyurethanes. The soft blocks (SS) in the bio-TPU’s materials were built from α,ω-oligo(ethylene-butylene adipate) diol. Hereby, bio-TPUs differed in hard segments content (c.a. 30; 34; 40, and 53%). We found that already increase of bio-based diisocyanate content of the bio-TPU impact the changes in their thermal stability which was measured by TGA. Based on DMTA results we observed changes in the viscoelastic behavior of bio-TPUs. The DSC analysis revealed decreasing in glass transition temperature and melting temperature of hard segments. In general, obtained materials were characterized by good mechanical properties. The results confirmed the validity of undertaken research problem related to obtaining bio-TPUs consist of bio-based hard building blocks. The application of partially bio-based diisocyanate mixtures and bio-based chain extender for bio-TPU synthesis leads to sustainable chemistry. Therefore the total level of “green carbons” increases with the increase of bio-based diisocyanate content in the bio-TPU structure. Obtained results constitute promising data for further works related to the preparation of fully bio-based thermoplastic polyurethane elastomers and development in the field of bio-based polymeric materials.

## 1. Introduction

Nowadays, in the production of new polymer materials, renewable or recycled substances are often used, especially in polyurethane synthesis. It is assumed that such materials will have at least similar or better properties compared to the origin one. The properties of polyurethanes (PUs) strongly depend on substrates used for their synthesis, monomers molar ratio, or processing methods. Because of their excellent properties and the possibilities of obtaining them with the usage of bio-based monomers, these materials are an issue in a lot of research papers. Special attention was given to PU materials synthesized using renewable sources such as vegetable oils and polysaccharides [1,2,3,4,5,6,7,8,9,10,11,12,13,14,15,16,17] or products of polyurethanes recycling known as recovered polyols obtaining in glycerolysis [18] or glycolysis [19,20,21] processes. Unfortunately, the application of alternative monomers does not always lead to producing polyurethane materials fully derived from environment-friendly monomers or materials with good mechanical properties. Typically in the structure of bio-based polyurethane materials is about 65% of petrochemical counterparts. The effective solution is introducing to the reactive PU systems, the optimal amount of bio-based or recovered monomers to maintain good mechanical or processing properties. The benefits that result from such proceedings are pro-environmental as well as economic. For instance, the pro-environmental benefit is related to relieving the environment from substrates that are produced in high energy consumption and emission processes. For instance, the pro-environmental benefit is related to relieving the environment from substrates that are produced in high energy consumption and emission processes. Biotechnological processes of natural oils or polysaccharides characterized lower emission CO_2_ to the atmosphere. The economical benefit is the reduction of the cost of conversion of biomass into monomer in comparison to the cost of crude oil refining [22].

Isocyanates, and also polyisocyanates next to polyols constitute the second main substrate used in polyurethane preparation. These monomers create a part of hard segments together with the low molecular weight of a chain extender or can be used as crosslinking agents. Isocyanates are highly reactive substrates that are caused by the presence of two cumulated unsaturated bonds, which provide different mesmeric structures. Commonly isocyanates are divided into aromatic and aliphatic. Their chemical structure and symmetry influence the course of the polymerization reaction and also future mechanical and physical properties of PUs, especially on thermal stability, tensile strength, Young modulus, hardness, or abrasion resistance. In general, the effect of diisocyanates structure on the properties of polyurethanes was studied by many researchers [23,24] for examples in terms of the determination of the viscoelastic properties of the polyurethanes [25] microphase separation [26,27,28].

Common examples of aromatic isocyanates are diphenylmethane diisocyanate (MDI), polymeric diphenylmethane diisocyanate (pMDI), and also 2,4–and 2,6- toluene diisocyanate (TDI). These aromatic isocyanates are mostly used in industry. For instance, commercial TDI consists of 80% of 2,4- isomers and 20% 2,6- isomers (TDI-80). It is worth mentioning that polyurethanes based on MDI are characterized by better tensile properties in comparison to polyurethanes based on TDI. In comparison to aliphatic isocyanates, aromatic isocyanates are more reactive what is caused by the possibilities of transfer of negative charge to the aromatic ring. Moreover, aromatic isocyanates are cheaper, have lower vapor pressure, and are usually used for polyurethane foams production. Unfortunately, polyurethanes obtained with aromatics isocyanates have poor resistance to UV radiation and, decomposed on aromatic amines which are toxic for living organisms.

Hexamethylene diisocyanate (HDI), isophorone diisocyanate (IPDI), bis(4-isocyanatocyclohexyl) methane (HMDI) belong to the common aliphatic diisocyantes. Polyurethanes synthesized using aliphatic isocyanates are applied to the coating and elastomers production. Because of good properties, biocompatibility, and UV resistance aliphatic isocyanates-based PU found application in medicine [29]. What is more, aliphatic isocyanates can be synthesized from renewable resources.

Despite the many advantages of the application of petrochemical-based diisocyanate, there are some disadvantages e.g., phosgene route of their synthesis, petrochemical origin of substrates, or high volatility. That is why the great interest in alternative environment-friendly methods for the preparation of bio-based diisocyanates has been observed in the last few years. Bio-based isocyanates and polymeric isocyanates are characterized by the significant value of weight equivalent in comparison to conventional counterparts, which is resulted from higher molecular weight substrates for their synthesis. This influence is revealed in the course of polymerization and also mechanical, thermal, physical, and processing properties, especially of thermoplastics polyurethane elastomers. On the other hand, polyurethanes obtained using fatty acids-based diisocyanates can display high elasticity or greater dumping properties.

Hitherto, there are known many environmentally friendly compounds used for obtaining of monomers for polyurethanes synthesis. For instance, as bio-based precursors for the synthesis of diisocyantes that natural oils (including fatty acids and their esters), amino acids, carbohydrates, fumaric derivatives, aromatic compounds, and aromatic compounds based on lignin. Some examples of bio-based and partially based diisocyanates that bio-based isocyanates-ethyl ester L-lysine diisocyanate (LLDI) and ethyl ester l-lysine triisocyanate (LLTI) are amino acids based diisocyanates; bio-based pentamethylene diisocyanate sugar-based diisocyanate; hydroxymethylfurfural; 2,4-diisocyanato-1-pentadecylbenzene based on cashew nut shell liquid and much more [30,31,32,33,34].

Promising green approaches in the development of new bio-based monomers for polyurethane production is connected with diisocyanates obtaining via a nonphosgene and green methods. More and coworkers [32] described the synthesis of fatty acid based diisocyanates via a non-phosgene route. As a starting compound for this purpose castrol oil derivatives were used. The preparation of bio-based diisocyanates such 1-isocyanato-10-[(isocyanatomethyl)thio]decane and 1,8-diisocyanatooctane was carried out via diacyl hydrazide intermediate. Obtained by Authors fatty acid based diisocyanates and few different diols were used for thermoplastic polyurethane preparation by a one-step solution polymerization method. Polyaddition reaction was carried out for 24 h, in anhydrous DMF, at 80 °C using DBTDL as a catalyst. The polyurethanes based on bio-based diisocyanates displayed semi-crystalline to amorphous behaviors featured by methylene dangling chains or cyclic moieties. These PUs materials decomposed typically in two step. The T10% values of PUs based on bio-diisocyanate was in the range 235–285 °C. Authors explained that this as result of higher hydrogen bonding density [32].

Next green monomers for TPUs synthesis were proposed by Charlon et al. [3]. Fully bio-based thermoplastic polyurethanes were obtained from isosorbide (molar mass = 146 g/mol), 2-heptyl-3,4-bis(9-isocyanatononyl)-1-pentylcyclohexane, 2-heptyl-3,4-bis(9-isocyanatononyl)-1-pentylcyclohexane (with molar mass = 587 g/mol) and 1,4-buthanediol renewable origin. The Authors used a dimerized acid-based diisocyanate, as a soft building block. Depends on the hard segments content bio-based TPUs were characterized by diverse macromolecular architecture. Thermal and mechanical properties were regulated by isosorbide and 1,4-butanediol. Increasing isosorbide contribution in TPUs structure caused poorer mechanical properties [3].

Li et al. studied the effect of polymerization method and utility of bio-based diisocyanate and chain extenders in crystalline segmented thermoplastic poly(ester-urethanes). TPUs were prepared by one-shot method or prepolymer method or multi-stage polyaddition method. The vegetable oil-based diisocyanate, 1,7-heptamethylene diisocyanate (HPMDI), poly(ethylene adipate) diol and bio-based glycols as chain extender (1,4-butanediol, 1,6-hexanediol, 1, 9-nonanediol and 1, 3-propanediol) have been used. Authors exhibited that materials obtained based on bio-based 1,7-heptamethylene diisocyanate possess toughness and strength comparable to those made from petroleum-based diisocyanates. The soft segment crystallinity might be changed and indicated on hard segments block structure [35].

In our previous work, the partially bio-based thermoplastic polyurethane elastomers (bio-PUs) were obtained using mixtures of aromatic–aliphatic, and aliphatic–aliphatic diisocyanate, polytetrahydrofurane (PolyTHF) and bio-1,3-propanediol (bio-PDO). Obtained bio-PUs characterized by reduction of 25 mass% of petrochemical diisocyanate (hexamethylene diisocyanate and diphenylmethane diisocyanate). TolonateTM X Flo 100 polyisocyanate was used as a bio-based diisocyanate which is a commercially available monomer, developed from palm oil. Obtained results led to conclude that application of mentioned bio-based diisocyanate has a positive impact on the thermal, thermomechanical, mechanical, and physicochemical properties of bio-PUs despite the diisocyanate type [36].

The research idea involved introducing the highest amount of bio-based monomers in the hard segment of the bio-based thermoplastic polyurethane elastomers synthesized of such partially bio-based diisocyanate and bio-based glycol (bioBDO). The aim was to obtain bio-based TPUs with remaining good mechanical properties and with potential for future processing. The influence of diisocyanate mixture used to obtain thermoplastic polyurethane elastomers, via a non-solvent method, on the chemical structure, thermal, thermomechanical, and mechanical properties was studied.

## 2. Materials and Methods

### 2.1. Samples Preparation

**Figure 1:**
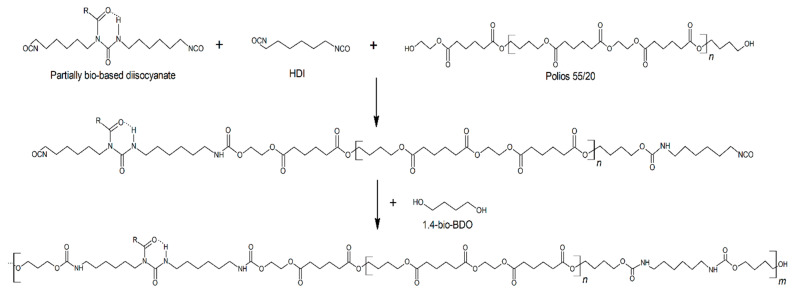
The scheme of synthesis of bio-based thermoplastic polyurethane elastomers via prepolymer method.

Bio-based thermoplastic polyurethane elastomers were obtained via the prepolymer method (Figure 1). Firstly, urethane prepolymers were synthesized from polyester polyol -Polios 55/20, Mn~2000 (Purinova, Bydgoszcz, Poland) diisocyanate mixture which contained 75/50/25 wt % of aliphatic bio-based polyisocyanate TolonateTM X Flo 100 (Vencorex Chemicals, Saint-Priest, France) and 25, 50, 75, and 100 wt % of hexamethylene diisocyanate HDI (Vencorex Chemicals, France), respectively. The reaction was conducted at 80 °C for 4 h. After prepolymerization, for each prepolymer, the free NCO content was determined by titration method according to ASTM D 2572-97. The free NCO was equaled 8.08%, 8.02%, 8.08%, and 8.06% for prepolymers contained 25, 50, 75, and 100 wt % of HDI in the diisocyanate mixture, respectively. During the second stage of the process, the prepolymer chains were extended by using bio-based glycol-1,4-butanediol (BASF, Ludwigshafen, Germany) with the presence of 0.2 wt % of dibutyltin dilaurate (DBTDL, Sigma Aldrich, Warszawa, Poland) as a catalyst. The polyurethanes were synthesized in the molar ratio [NCO]prepolymer/[OH]chain extender equaled 0.95, 1.0, 1.05, and 1.1. The bio-based thermoplastic polyurethane elastomers were molded and cured at 100 °C for 24 h in a laboratory oven to obtain fully crosslinked materials.

### 2.2. Measurement

#### 2.2.1. Fourier Transform Infrared Spectroscopy—FTIR

FTIR-ATR technique was used for the chemical structure analysis of bio-based thermoplastic polyurethane elastomers obtained in [NCO]/[OH] molar ratio equaled 1.0 by identification of characteristic chemical groups of polyurethanes. Measurements were taken by using FTIR Nicolet 8700 spectrophotometer (Thermo Electron Corporation, Waltham, MA, USA) equipped with Specac Heated Golden Gate, single reflection diamond ATR accessory. Each spectrum was registered at room temperature for the standard wavenumbers ranging from 500 to 4500 cm^−1^ at 4 cm^−1^ nominal resolution. FTIR spectroscopy was used to determine the degree of phase separation (DPS) as R/(R + 1), where R is a carbonyl hydrogen bonding index and expressed as a ratio of absorption intensity of hydrogen-bonded carbonyl and absorption intensity of free carbonyl. Before peaks deconvolution, spectra’ were normalized.

#### 2.2.2. Differential Scanning Calorimetry—DSC

Differential scanning calorimetry was performed by using DSC 204 F1 Phoenix^®^ calorimeter (NETZSCH, Selb, Germany). Each sample with a weight of ca. 10 mg was placed in a closed aluminum pan and heated twice from −80 °C to 240 °C at a scanning rate of 10 °C/min and cooled once from 240 °C to −80 °C at a scanning rate of 5 °C/min. Measurements were taken using N_2_ as a purge gas (20 mL/min). The endothermic curves were used for the determination of glass transition temperature (T_g_), melting temperature (T_m_), and melting enthalpy (ΔH_m_), for both, the first and second run. Based on the exothermic curve, the crystallization temperature (T_c_), and crystallization enthalpy (ΔH_c_) were determined. DSC analysis was conducted on the bio-TPU samples obtained in [NCO]/[OH] molar ratio equaled 1.0.

#### 2.2.3. Dynamic Mechanical Analysis—DMA

Dynamic mechanical analysis of bio-TPU obtained in [NCO]/[OH] molar ratio equaled 1.0 was performed according to ASTM D4065:2012 using a DMA Q800 Analyzer (TA Instruments, New Castle, DE, USA) under nitrogen atmosphere. The measurements were made at a temperature range from −100 to +120 °C, at an operating frequency of 10 Hz. The heating rate was 4 °C/min. The dimension of rectangular samples was ca. 18 × 10 mm, and a thickness of 3 mm. The values of storage modulus (E′), loss modulus (E″), and glass transition temperature of soft segments, T_gSS_ (based on the dependence of tanδ upon temperature) were also determined.

#### 2.2.4. Thermogravimetric Analysis—TGA

Thermogravimetric analysis (TGA) was carried out using a Perkin Elmer instrument as Pyris 1 (Perkin Elmer, Waltham, MA, USA). The measurements were performed under N_2_ atmosphere, at a heating rate of 20 K/min, and temperature range from 35 to 650 °C. The results of the TGA analysis were presented as the TG and DTG curves.

#### 2.2.5. X-ray Diffraction (XRD)

X-ray diffraction (XRD) was conducted by Phillips X’Pert Pro diffractometer (XRD, Panalytical, Almelo, Netherlands) with CuKα radiation (1.540 Å). The measurements were performed at 40 kV and 30 mA at room temperature. The diffraction angle of 2θ was ranged from 10° to 60°.

#### 2.2.6. Tensile Test

The tensile strength (T_Sb_) and elongation at break (E_b_) were measured under static condition by using a Zwick Roell tensile-testing machine (Zwick Z020, Zwick Roell Group, Ulm, Germany), in accordance with the EN ISO 527-1:1996 and EN ISO 527-2:1996 standards. The dumbbell-shaped samples of standard dimensions were tested. The original gauge length (lo) was equaled 25 mm. The measurements were performed at a rate of 100 mm/min. Measurements were taken at room temperature.

#### 2.2.7. Hardness

Hardness was measured according to the PN-EN ISO 868:2005 standard. The circular samples were placed on a flat surface, and 10 measurements were taken per sample by applying a Shore A durometer (Zwick Roell Group, Ulm, Germany) for 3 s.

## 3. Results and Discussion

### 3.1. FT-IR Spectroscopy Analysis

**Figure 2:**
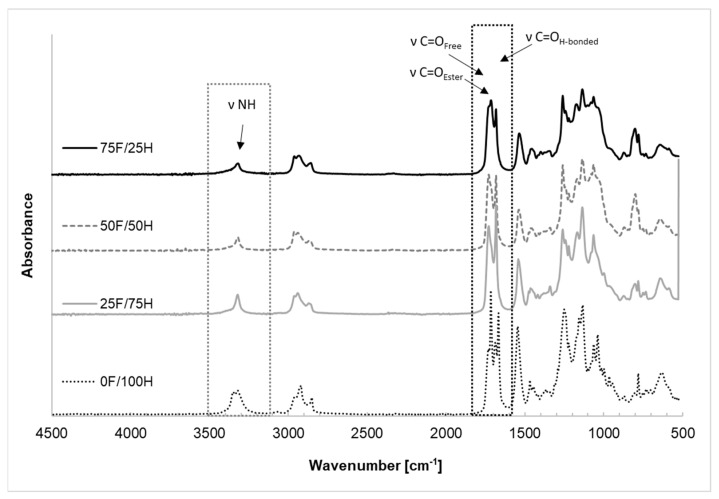
FTIR-ATR spectra of bio-based thermoplastic polyurethane elastomers containing different amount of bio-based diisocyanate.

**Figure 3:**
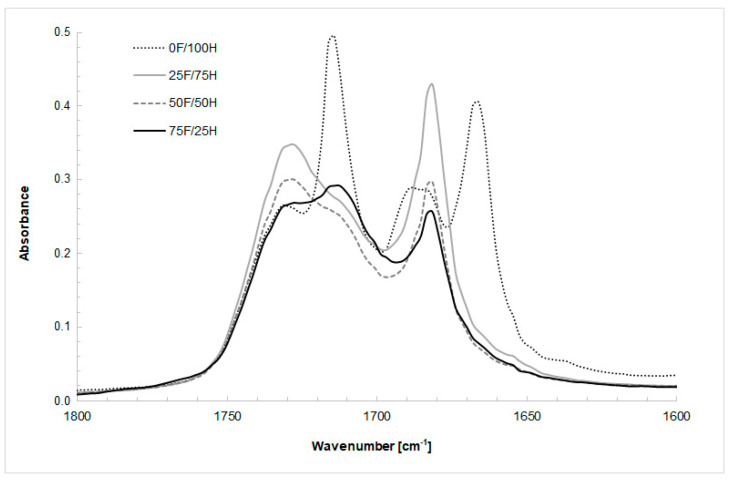
FTIR-ATR spectra at the range of wavenumber of carbonyl groups derived from bio-based thermoplastic polyurethane elastomers containing different amount of bio-based diisocyanate.

**Figure 4:**
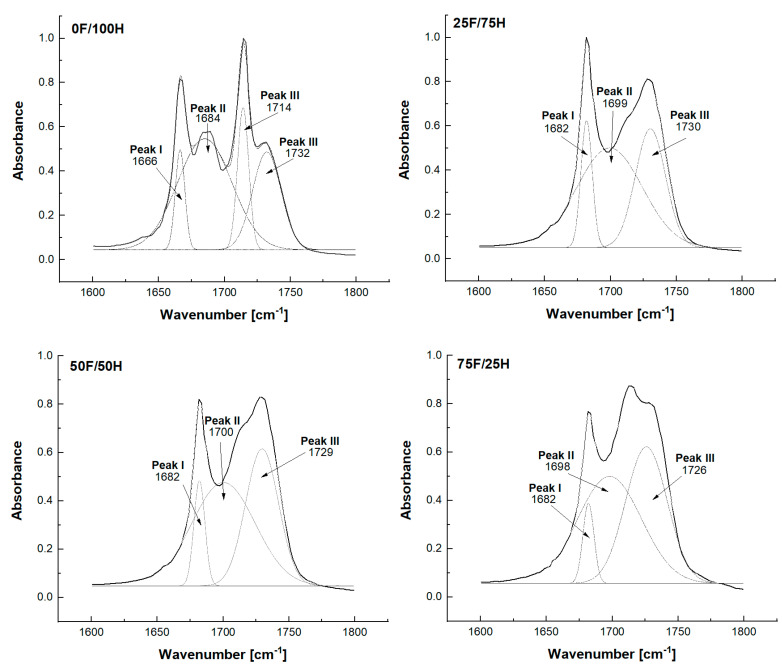
Deconvoluted peaks of absorption at the range of stretching vibration of carbonyl group presented in the structure of bio-based thermoplastic polyurethane elastomers containing different amount of bio-based diisocyanate.

**Figure 5:**
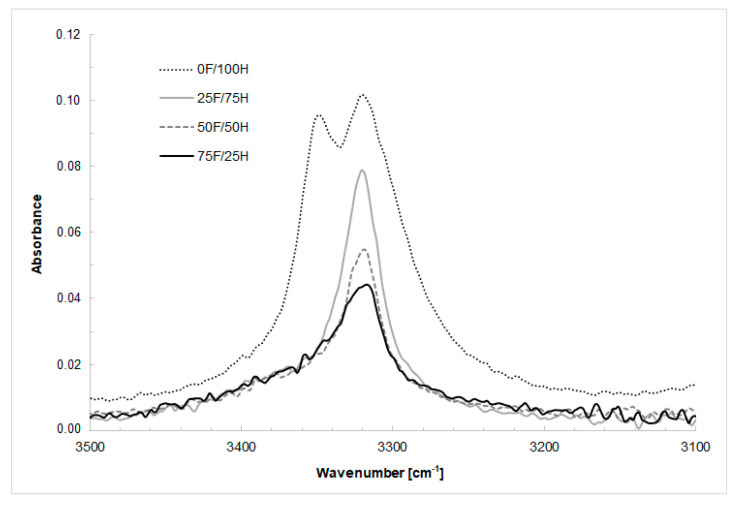
FTIR-ATR spectra at the range of NH bond wavenumber derived from urethane groups of bio-based thermoplastic polyurethane elastomers containing different amount of bio-based diisocyanate.

Chemical structure and the changes in the range of signal of the urethane groups, present in the bio-based thermoplastic polyurethane elastomers, were recorded by FTIR spectroscopy and the spectra are shown in Figure 2, Figure 3, Figure 4 and Figure 5. What is more, the phase separation in polyurethane materials and its self-segregation in hard segments were also determined by the FTIR technique. For this purpose, the intensities and positions of the hydrogen-bonded (H-bonded) and free hydrogen-bonded carbonyl groups, and N-H stretching vibrations were investigated [37].

All FTIR analysis confirmed, in the spectra (Figure 2) at the range of wavenumber 2270 cm^−1^ there did not assign the vibration derived from free –NCO groups what suggests fully reacted monomers and correct conditions of polymerization. In general, at the spectra of bio-based thermoplastic polyurethane elastomers were identified vibration derived from –NH, –C(O)O, –C=O, –CN, –CH, –CH_2_, –CH_3_ groups. Regardless of the bio-TPU type, the stretching vibration of CH_2_ groups is visible at the wavenumbers of 2854 cm^−1^ and 2918 cm^−1^ (asymmetric and symmetric) as well as 1412 cm^−1^ (deformation vibrations). Ester group derived from polyol component, as stretching vibration of –C(O)O group, occurred as a multiple vibration at 1130 cm^−1^ and 1038 cm^−1^. Changes in the structure of bio-based thermoplastic polyurethane elastomers were observed in the range of vibration of –C=O and –NH groups.

Analyzing the carbonyl bond of polyurethane materials it is important to take into account its complexity. The peak in the range of carbonyl bond vibration mainly consists of free carbonyl groups (from soft segments), free hydrogen-bonded, and H-bonded urethane carbonyl groups derived from hard segments. In this region, we can also consider H-bonded carbonyl groups created between soft-hard segments. The position of H-bonded urethane carbonyl groups can indicate disordered (amorphous regions) or order phase in hard segments (regions of order or some regularity in the structure). Thus the number of hydrogen-bonded carbonyls can be related to the extent of hard segment bonding in hard domains. Phase separation is higher if hydrogen bonds exist only within the hard segment domains. Lower phase separation occurred in the case of the interphase hydrogen bonding presence, between the soft and hard segments [38,39].

According to this knowledge, a profound study on the changes in the range of carbonyl group vibration was conducted. First of all, the differences in band intensity and peak shifts at the region of NH and C=O were observed and depended on the diisocyanates composition ratio. The stretching vibration of carbonyl group of obtained bio-TPUs occurred as a multiple peak at a wavenumber range from 1600 cm^−1^ to 1730 cm^−1^. The exact position of peaks depends on the amount of bio-based diisocyanate used for preparation of diisocyanate mixture. Considering the reference sample (0F/100H) synthesized without bio-based diisocyanate content, the peak of stretching vibration of H-bonded C=O in ordered crystalline hard domains was assigned at 1666 cm^−1^ while for the disordered phase at 1714 cm^−1^ (Figure 3). For the samples contained bio-based diisocyanate, the peak related with hydrogen bonds in the hard segments (in the ordered phase) was assigned at 1680 cm^−1^ and absorption peak of disordered hydrogen-bonded carbonyl groups revealed at 1712 cm^−1^. The free carbonyl of urethane groups, and also residue of polyester based polyol in all cases were assigned at 1730 cm^−1^. Based on the FTIR results and deconvolution technique of carbonyl peaks, the carbonyl index (R), the degree of phase separation (DPS), the degree of phase mixing (DPM) were calculated and presented in Table 1. The deconvoluted peaks were shown in Figure 4.

The hydrogen bonding index (R), in the polyurethanes, was calculated as a ratio of absorption intensity of hydrogen-bonded carbonyl, and absorption intensity of free carbonyl from the FTIR spectra. The degree of phase separation (DPS) was determined using the equation: DPS = R/(R + 1). According to the literature, with R increasing, the degree of phase separation in polyurethanes increase [40]. It was observed that the degree of phase separation of obtained bio-TPU depends on the content of the hard segment, and the same, on the amount of bio-based diisocyanate in the structure. With the increasing of bio-monomers in the structure of hard segments, the DPS decreased what indicated of the deteriorated phase separation. The highest value of DPS, 0.780 was noticed for reference materials (0F/100H) what can be related with the linear structure of 1,6 hexamethylene diisocyanate.

The stretching vibration of NH groups was assigned as multiple peaks for reference material at the range of 3346 cm^−1^ (non-hydrogen-bonded NH) and 3319 cm^−1^ (hydrogen-bonded NH) [14], and as a single peak for bio-based thermoplastic polyurethane elastomers based on diisocyanate mixture. Based on FTIR spectra, given in Figure 5, at the range of NH bond wavenumber derived from urethane groups of bio-TPUs containing different amount of bio-based diisocyanate it can be observed that the intensity of vibration bands strongly depend on the amount of bio-based diisocyanate in the structure of bio-TPU. At the range of wavenumber 1540 cm^−1^ and 1530 cm^−1^, the vibration of NH (out-of-plane bending vibration) and CN (stretching vibration) groups were identified. Their position depends on the amount of amount of the bio-based diisocyanate. NH out-of-plane wagging vibrations were assigned at the range of wavenumber from 650 to 900 cm^−1^.

### 3.2. Thermogravimetric Analysis

**Figure 6:**
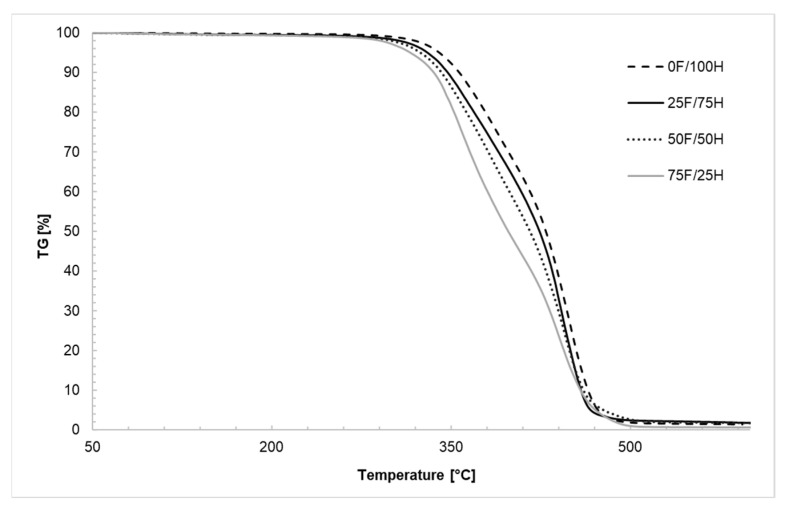
TG curves of bio-based thermoplastic polyurethane elastomers containing different amount of bio-based diisocyante and reference sample without bio-based diisocyanate.

**Figure 7:**
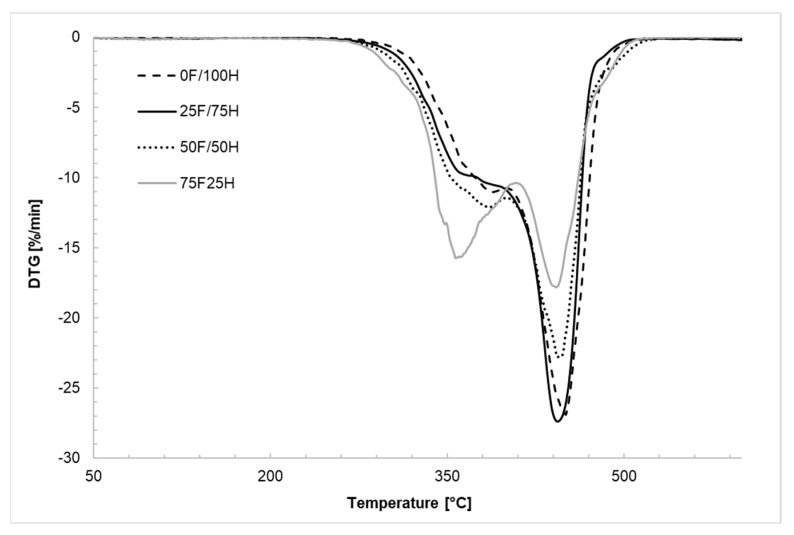
DTG curves of bio-based thermoplastic polyurethane elastomers containing different amount of bio-based diisocyanate.

Thermal stability of obtained bio-based thermoplastic polyurethane elastomers was determined by thermogravimetric analysis. For analysis, the bio-TPU synthesized in [NCO]/[OH] molar ratio equaled 1.0 were used. In Figure 6 and Figure 7, the TG and DTG curves were presented while in Table 2 the temperatures of 5%, 10%, and 50% weight loss and ash residue at 600 °C were shown. Based on conducted measurements it was noticed that polyurethanes synthesized using diisocyanate mixture degraded in two independent steps regardless of the amount of bio-based diisocyanate used for preparation diisocyanate mixture. This is a suspected observation in the case of polyurethane materials. In the first stage of the thermal decomposition, the hard segments degraded, while the second stage of mass loss is correlated with the decomposition of soft segments [41,42]. An increase in the amount of bio-based diisocyanate in the diisocyanate mixture lead to a slight decreasing in the beginning of the thermal degradation (Figure 6), and increasing in degradation speed (Figure 7). It is visible that an increase of bio-based diisocyanate amount caused shifting of the beginning of degradation to the lower temperature about 6–9 °C (Table 2). Decreasing in thermal stability probably is caused by decreasing in the ordering of the hard segments and increasing in amorphous phase [29] what is also confirmed by DSC result, as a decrease in crystallization temperature. The structure of partially bio-based diisocyanate, used in the diisocyanate mixture, has also influenced these changes. The presence of residue of fatty acid, in the side chain, as a steric hindrance caused the intermolecular forces to decrease, and thermal dissociation of urethane groups occurred in the lower temperature. It might be also a reason for the increase in speed of the degradation in the case of materials synthesized with a higher share of bio-based diisocyanate. Nevertheless, comparing thermal properties of obtained bio-TPUs with materials based on bio-based diisocyanates which are described in the literature [32] it was observed that they are characterized by higher T_10%_ about 80 °C.

### 3.3. Differential Scanning Calorimetry

**Figure 8:**
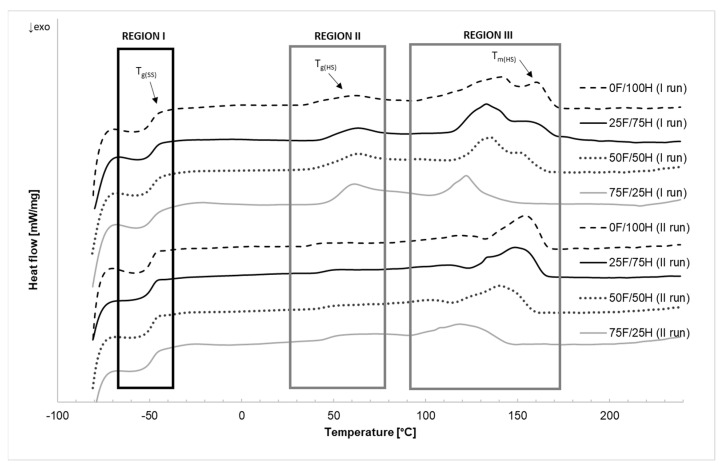
The endothermic curves (first (I) and second (II) run) of bio-based thermoplastic polyurethane elastomers containing different amount of bio-based diisocyanate.

**Figure 9:**
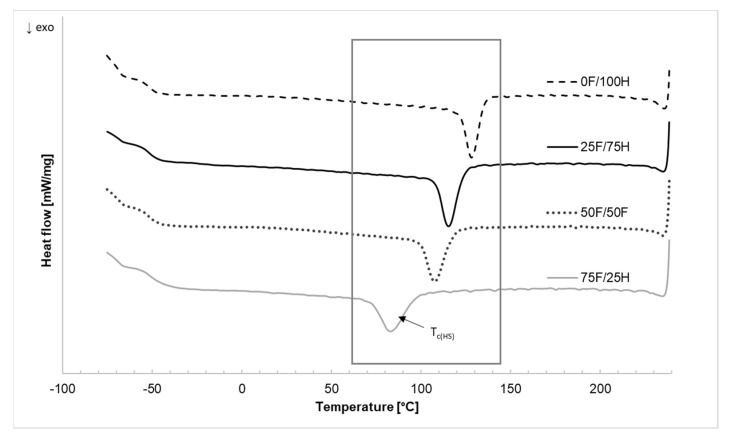
The exothermic curves of bio-based thermoplastic polyurethane elastomers containing different amount of bio-based diisocyanate.

The effect of bio-based diisocyanate amount used in the bio-based thermoplastic polyurethane elastomers preparation is strongly revealed in the results of differential scanning calorimetry and the changes that occurred in the range of hard segments. The results of DSC measurements were shown in Table 3 and Figure 8 and Figure 9.

The endothermic curves (Figure 8) present two heating runs which were divided into three main areas. The first region, at the lowest temperature range, is correlated with the glass transition of soft segments (T_gSS_) of bio-TPU. The second, and third regions were attributed to the hard phase, exactly to the glass transition of hard segments and their melting point.

At the first region of DSC endothermic curves, the glass transition temperature of soft segments (T_gSS_), irrespective of bio-based diisocyanate content, and heating run was analyzed. For each of bio-based thermoplastic polyurethane elastomers, the T_gSS_ was recorded as ca. −49 °C. At the second region of the curves, depending on the heating run, the glass transition temperature of hard segments (T_gHS_) for each tested material was assigned and equaled about 53 °C (I run) and 47 °C (II run). At the endothermic curves which represented the first heating run (I run), the multiple peaks were observed at the temperature range from 100 to 170 °C. It has resulted from differences in the size of the hard microdomains, theirs amounts, and also depended on the thermal history of the bio-based thermoplastic polyurethane elastomers what confirmed literature [43]. The melting temperature (T_mHS_) of hard segments depends on the diisocyanate mixture type and with increasing bio-based diisocyanate content, the T_mHS_ decreased. Moreover, the bordering of the peaks (first heating run) was observed, which can suggest some inhomogeneity in hard segments caused by the disordering of the hard microdomains and also fraction differences in molecular weight. After cooling and repeated heating, in the second heating run curves (run II) the influence on the formation of more ordered structures in the hard phases were releveled as single peaks. Especially it is visible for reference sample (coded 0 F/100 H) as a good shape narrow peak with the highest value of enthalpy of melting. What is more decreasing in peak area with an increase of bio-based diisocyanate share in the TPU structure indicated on the formation of the lower part of an ordered phase in comparison to reference of bio-TPU samples. Bio-based polyurethanes obtained with a higher amount of linear petrochemical diisocyanate (HDI) tend to polymer chain to the formation of ordered structure. It is well known that the ability to the ordering of hard segments increases with the increasing of symmetry of diisocyanate.

Analyzing the exothermic curves of bio-based thermoplastic polyurethane elastomers containing different amounts of bio-based diisocyanate decrease in crystallization temperature was observed. The higher content of bio-based diisocyanate the lower T_cHS_ and higher enthalpy (Figure 9). This observation suggests that bio-based diisocyanate caused decreasing in structure order and crystalline phase content. Growth in the molecular weight of hard segments eventuated from increasing of bio-based diisocyanate amount characterized by higher molecular weight and with the presence of side-chain might cause limitation in self-organization of macromolecular chains in the ordered phase. Part of hard segments is dispersed in soft phase, and some parts can create hard segments domain in the hard phase.

### 3.4. Dynamic Mechanical Analysis

**Figure 10:**
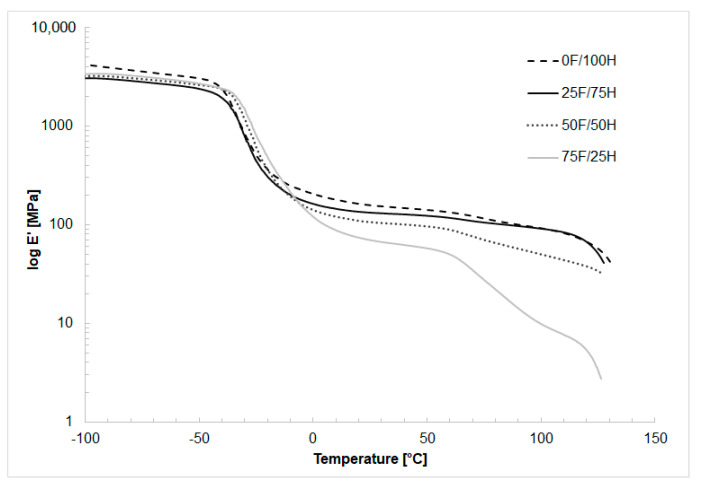
The storage modulus (log E′) as a function of temperature thermoplastic polyurethane elastomers containing different amount of bio-based diisocyanate.

**Figure 11:**
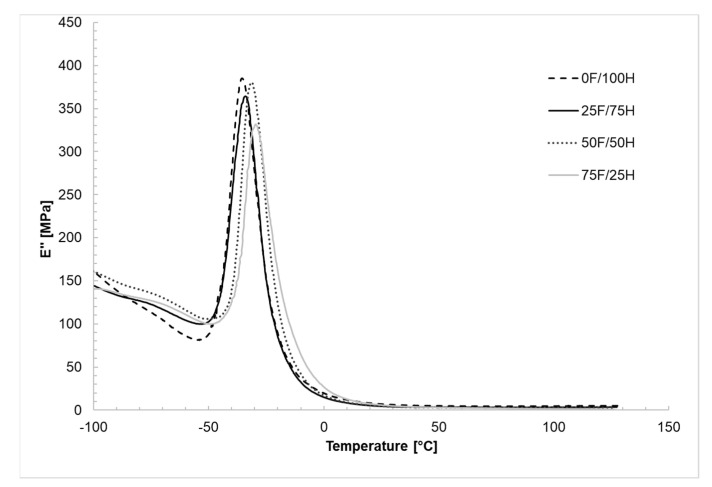
The loss modulus (E″) as a function of temperature thermoplastic polyurethane elastomers containing different amount of bio-based diisocyanate.

**Figure 12:**
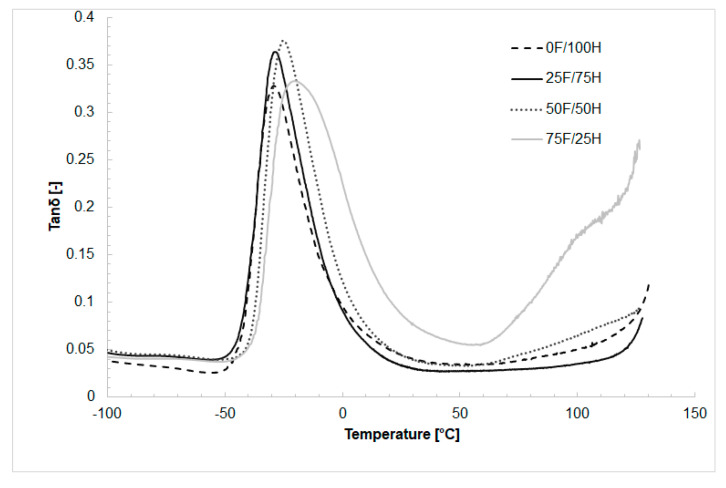
The tanδ as a function of temperature thermoplastic polyurethane elastomers containing different amount of bio-based diisocyanate.

In Figure 10, Figure 11 and Figure 12 the results of thermal dynamical analysis have been shown. The influence of the amount of bio-based diisocyanate in the diisocyanates mixture on the storage (E′) and loss modulus (E″), as well as tanδ, was determined. The analysis was performed on the bio-TPU obtained in [NCO]/[OH] molar ratio equaled 1.0.

The bio-based thermoplastic polyurethane elastomers characterized the higher value of storage modulus at the temperature below glass temperature of soft segments (ca. −35 °C) and exactly at range of the γ-transition of methylene groups was registered for reference material. At room temperature (25 °C) also reference material had the highest storage modulus (157 MPa). Moreover with an increase in the amount of bio-based diisocyanate mentioned modulus decreased. What is more, analyzing the dependence of storage modulus upon temperature, the significant changes of viscoelastic behavior is noticed, especially in the range of higher temperature, above the glass transition of hard segments, 60 °C (Figure 10). High amount of bio-based diisocyanate caused softening in lower temperatures. This result confirmed decreasing in order in hard segments and increasing in the amorphous phase. These results are consonant with DSC analysis described earlier.

The loss modulus E′′ curves were presented in Figure 11. A small peak at the range above −50 °C was assigned to the γ-transition of methylene groups that occurred in the structure of synthesized materials and corresponded to their local motions. Well-shaped peaks were registered in the range of temperature from −50 to 0 °C with the maximum value of loss modulus equaled 384 MPa and assigned for reference material. What is more, based on loss modulus curves it can be concluded that more mechanical energy is dissipated as heat in the case of samples with lower content of bio-based diisocyanate in the structure.

The glass transition temperature of soft segments (T_gSS_) determined based on tangent δ curves was in the range of −35 to −19 °C, and increased with increasing the amount of bio-DIISO (Figure 12). The loss factor (tanδ) for each material was similar, nevertheless, materials obtained with a higher amount of bio-based diisocyanate are characterized by poorer damping properties. In Figure 10 and Figure 12, the glass transition temperature of hard segments (T_gHS_) is visible above 60 °C.

### 3.5. X-ray Diffraction (XRD)

**Figure 13:**
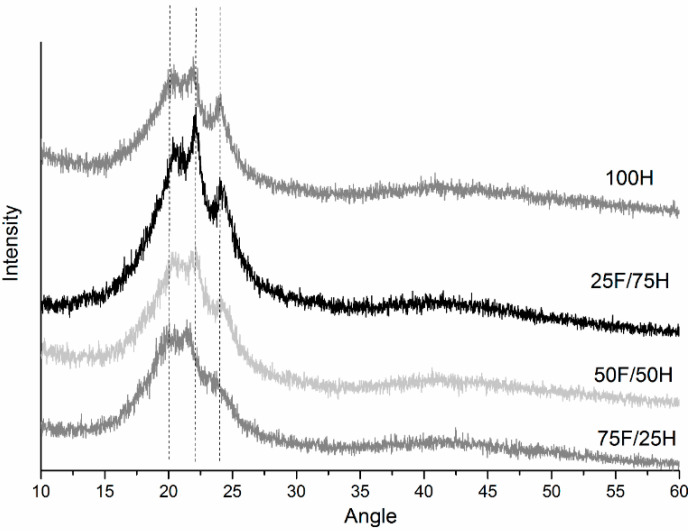
X-ray diffractograms of thermoplastic polyurethane elastomers containing different amount of bio-based diisocyanate.

XRD analysis was performed to support the DSC results which indicated a degree of ordering in the prepared bio-TPUs materials and confirm the phase separation between hard and soft segments. XRD patterns were presented in Figure 13 with the main diffraction signals at 2θ for 25 F/75 H 20.4°, 22.1°, 24.1°; 50F/50H at 21.4°, 23.7°, and for 75F/25H 20.4°, 21.4°, 22.1° and they correspond to the crystalline form, derived from hard and soft segments, what is accordant with the literature [44]. The XRD obtained patterns indicate that all the samples showed partially crystalline structures which get lowered with increasing of bio-based diisocyanates content in the diisocyanate mixture what is in agreement with the results of DSC analysis.

### 3.6. Tensile Properties

The possibility to apply polymeric materials to different purposes is determined by their mechanical properties. That is why the tensile properties such as tensile strength TS_b_ and elongation at break E_b_ were examined. The results of the tensile test were shown in Table 4.

It is known that the mechanical properties of polyurethane depend on the hard segment content, order in the hard phase and symmetry of the diisocyanate [45]. Based on obtained results the strong influence of the amount of bio-based diisocyanate was observed (Table 4). Special attention was given for bio-TPU samples synthesized in [NCO]/[OH] molar ratio equaled 1.0 which tensile strength increased with increasing of bio-based diisocyanate content up to 50 wt %, and it can be concluded that this is the optimum amount of bio-based diisocyanate in the diisocyanate mixture which allow obtaining materials with good mechanical characteristic.

Decreasing in tensile strength and a slight increase in hardness in the case of sample coded 75F/25H (with the highest amount of hard segments) can be the result of the phase inversion. According to the literature, above certain HS content (40% for PU based on MDI, PTMG, and BDO), hard phase becomes continuous phase, and the increase in hardness of polyurethanes is observed [45]. The values of hardness for thermoplastic polyurethane elastomers containing the different amount of bio-based diisocyanate and synthesized in [NCO]/[OH] molar ratio equaled 1.0. were change and equaled 90.8 ± 0.7°ShA; 83.8 ± 1.4°ShA; 83.9 ± 1.4°ShA for samples coded 25F/75H, 50F/50H, and 75F/25H, respectively.

The effect of [NCO]/[OH] molar ratio on the tensile properties was examined too (Table 4). According to theory [43,45], with increasing of [NCO]/[OH] molar ratio the tensile strength increased. The addition of the bio-based diisocyanate to the synthesis of bio-TPU and the partial replacement of petrochemical diisocyanate caused increasing in elongation at break. This phenome can result from the partially bio-based diisocyanate structure and the presence of fatty acids that can act as a plasticizer.

## 4. Conclusions

Novel bio-based thermoplastic polyurethane elastomers were synthesized using diisocyanate mixture from partially bio-based and petrochemical diisocyanates and polyester-based polyols, and bio-based 1,4 butanediol. The bio-TPUs were synthesized via the pre-polymer method. Polyurethane samples were prepared at four molar ratios of NCO and OH groups equaled 0.95, 1.0, 1.05, and 1.1. The advantage of the proposed materials is the fact that they are obtaining by the free solvent method and with high content of green carbon in the structure of hard segments what is consistent with sustainable chemistry. The content of partially bio-based isocyanate was 25, 50, and 75 wt %, respectively. Reference material, without the addition of bio-based diisocyanate, was obtained and investigated, too. Based on recorded results it was found that increasing of bio-based diisocyanate in the diisocyanate mixture impact strongly on thermal and thermomechanical properties of bio-TPU. We conclude that these changes are caused by changes in the order in hard segments what is a consequence of the difference at phase separation of hard and soft segments.

Based on FTIR analysis it was found that the degree of phase separation depends on the total content of hard segments, and decreased with the increasing bio-based diisocyanate content in the bio-TPU. We noticed that increasing of bio-based diisocyanate slightly decreased thermal stability of bio-TPU (from 339 °C for reference material (0F/100H) to 316 °C for bio-TPU with the highest amount of bio-based diisocyanate (75F/25H)). Nevertheless thermal characteristic is better than similar of bio-based polyurethane materials obtained by other researches what is known from the literature. DSC results indicated that the glass temperature of soft segments for all bio-TPU’s materials is ca. −49 °C while the glass temperature of hard segments is below 60 °C and depends on the content of HS. What is more, the melting and crystallization temperature of hard segments also decreased within a specified range of values. The XRD obtained patterns indicate that all the bio-TPU samples showed partially crystalline structures. It seems that bio-based diisocyanate content of about 50 wt % is the optimum amount of bio-based diisocyanate in the diisocyanate mixture which allows obtaining materials with good mechanical properties (with TS_b_ from 6.5 to 33.3 MPa).

Taking into account thought good thermal and mechanical characteristics of obtained thermoplastic polyurethanes they would be used in the 3D printing, injection molding, or extrusion processes at similar conditions to these which are known from the literature. The proposed route of TPU synthesis can show a useful greenway with the possibility of changing hard segment blocks as needed in the planned application.

## Figures and Tables

**Table 1:** The carbonyl index (R), the degree of phase separation (DPS), the degree of phase mixing (DPM), and hard segments content (wt % HS) of bio-based thermoplastic polyurethane elastomers.

Parameter	Bio-Based Diisocyanate Content (wt %)			
	0	25	50	75
R	3.56	1.92	1.56	1.43
R^2^	0.9931	0.9976	0.9963	0.9943
DPS	0.780	0.657	0.609	0.588
DPM	0.220	0.343	0.391	0.412
HS [%]	29.6	34.0	40.2	53.4

R-carbonyl index; R^2^-coefficient of determination.

**Table 2:** Thermal properties of bio-based thermoplastic polyurethane elastomers determined by thermogravimetry.

Properties	Bio-Based Diisocyanate Content (wt %)			
	0	25	50	75
T_5%_ [°C]	339	330	324	316
T_10%_ [°C]	357	347	341	335
T_50%_ [°C]	429	424	415	398
Ash residue at 600 °C [%]	1.4	1.7	1.7	0.6

**Table 3:** Thermal properties of bio-based thermoplastic polyurethane elastomers determined by DSC technique.

Properties	Bio-Based Diisocyanate Content (wt %)							
	0		25		50		75	
	I Run	II Run	I Run	II Run	I Run	II Run	I Run	II Run
T_gSS_ [°C]	−49.5	−49.1	−48.9	−49.1	−49.2	−48.9	−49.7	−49.1
T_gHS_ [°C]	43.9	38.0	51.8	44.2	54.0	44.7	54.2	47.1
T_mHS_ [°C]	160.2	154.1	161.2	148.7	150.7	140.3	122.1	118.4
ΔH_mHS_ [J/g]	16.67	12.34	20.68	13.27	16.19	11.92	5.34	10.01
T_cHS_ [°C]	128.2	-	115.4	-	107.7	-	83.1	-
ΔH_c_ [J/g]	−11.39	-	−14.82	-	−13.78	-	−14.62	-

**Table 4:** The values of TS_b_ and E_b_ for thermoplastic polyurethane elastomers obtained with different [NCO]/[OH] molar ratio from 0.95 to 1.1.

Properties	The Diisocyanate Mixture Type			
	0F/100H			
[NCO]/[OH]	0.95	1.0	1.05	1.1
TS_b_ (MPa)	9.2 ± 0.6	16.8 ± 2.8	17.9 ± 2.6	25.8 ±4.5
ε_b_ (%)	218 ± 67	706 ± 139	613 ± 82	803 ± 86
	25F/75H			
[NCO]/[OH]	0.95	1.0	1.05	1.1
TS_b_ (MPa)	10.8 ± 0.8	25.7 ± 1.9	29.8 ± 1.9	31.7 ± 2.2
ε_b_ (%)	449 ± 98	883 ± 52	890 ± 16	874 ± 29
	50F/50H			
[NCO]/[OH]	0.95	1.0	1.05	1.1
TS_b_ (MPa)	8.7 ± 0.5	27.5 ± 3.1	24.9 ± 8.3	33.3 ± 1.8
ε_b_ (%)	449 ± 102	895 ± 16	809 ± 91	904 ± 15
	75F/25H			
[NCO]/[OH]	0.95	1.0	1.05	1.1
TS_b_ (MPa)	6.5 ± 0.6	16.1 ± 0.9	18.9 ± 0.9	18.4 ± 1.2
ε_b_ (%)	421 ± 4	818 ± 41	845 ± 40	883 ± 43

## Data Availability

Data are contained within the article. The data presented in this study are available in The Green Approach to the Synthesis of Bio-Based Thermoplastic Polyurethane Elastomers with Partially Bio-Based Hard Blocks.
